# Protective Effect of Peptides from *Pinctada Martensii* Meat on the H_2_O_2_-Induced Oxidative Injured HepG2 Cells

**DOI:** 10.3390/antiox12020535

**Published:** 2023-02-20

**Authors:** Jie Zhou, Mengfen Wei, Lijun You

**Affiliations:** 1 School of Food Science and Engineering, South China University of Technology, Guangzhou 510641, China; 2 Guangzhou Institute of Modern Industrial Technology, Guangzhou 511458, China

**Keywords:** *Pinctada martensii*, hydrolysis, antioxidant peptides, purification, peptide synthesis

## Abstract

*Pinctada martensii* is a major marine pearl cultured species in southern China, and its meat is rich in protein, which is an excellent material for the preparation of bioactive peptides. In this study, the peptides from *Pinctada martensii* meat were prepared by simulated gastrointestinal hydrolysis, and after multistep purification, the structures of the peptides were identified, followed by the solid-phase synthesis of the potential antioxidant peptides. Finally, the antioxidant activities of the peptides were verified using HepG2 cells, whose oxidative stress was induced by hydrogen peroxide (H_2_O_2_). It was shown that the antioxidant peptide (S4) obtained from *Pinctada martensii* meat could significantly increase the cell viability of HepG2 cells. S4 could also scavenge reactive oxygen species (ROS) and reduce the lactate dehydrogenase (LDH) level. In addition, it could enhance the production of glutathione (GSH) and catalase (CAT) in HepG2 cells, as well as the expression of key genes in the *Nrf2* signaling pathway. Three novel antioxidant peptides, arginine–leucine (RL), arginine–glycine–leucine (RGL), and proline–arginine (PR), were also identified. In conclusion, peptides from *Pinctada martensii* meat and three synthetic peptides (RGL, RL, PR) showed antioxidant activity and could have the potential to be used as antioxidant candidates in functional foods.

## 1. Introduction

Reactive oxygen species (ROS), which act as the signal molecule in the metabolic oxidative/antioxidant balance of organs, are often generated in the aerobic respiratory electron transport chain [1,2]. However, when an imbalance appears between the generation and the elimination of ROS under pathological conditions, the accumulation of ROS causes oxidative stress on cell metabolism [3]. It is reported that the development of many diseases, such as heart disease, gastrointestinal inflammation, and cancer, are linked to the ROS-induced oxidation of proteins, polysaccharides, and lipids [2,4,5,6]. To avoid oxidative damage, cells possess various antioxidative defense effects, such as enzymatic and nonenzymatic antioxidant systems, for eliminating ROS. Furthermore, it has been determined that the *Nrf2* signaling pathway is critical for enhancing antioxidation by increasing the expression of related-antioxidant enzymes [7,8]. Furthermore, it has been found that natural antioxidants could effectively prevent the damage that ROS causes. As a result, studies about various natural antioxidant compounds, such as peptides prepared from natural proteins [9,10,11,12], are becoming increasingly popular [2].

Generally, enzymatic hydrolysis is the most common and well-accepted safe method for preparing bioactive peptides. So far, commercial enzymes such as Alcalase, neutral protease, and papain have been widely applied in preparing antioxidant peptides from seafood such as cuttlefish [13], whiting fish [14], and tuna [15]. Considering the fact that peptide bioavailability could affect their applicability since peptide structure could change in the gastrointestinal tract due to pH change and the action of digestive enzymes during digestion and absorption, simulated gastrointestinal hydrolysis has been utilized to hydrolyze protein in some studies [16]. The above enzymatic method, including two-stage enzymatic hydrolysis, improves the bioavailability and production of low molecular weight peptides by sequentially simulating digestion using pepsin, trypsin, and chymotrypsin in vitro [1].

*Pinctada martensii*, a major cultured seawater pearl species, is widely cultured in southern China, particularly in the provinces of Hainan, Guangdong, and Guangxi. The pearls produced by the species with an annual pearl yield is 15–30 t [17]. Take Guangxi Province as an example. The annual output of *Pinctada martensii* meat (all organs) can reach 2000–3000 t [18]. After the pearling process, the meat from *Pinctada martensii* is typically discarded without further utilization, causing a lot of waste [19]. Like many seafood, *Pinctada martensii* meat is rich in high-quality proteins and essential amino acids, which suggests it would be a potential material for preparing bioactive peptides [20]. Xia et al. have produced collagen peptides with antioxidant activity from *Pinctada martensii* meat using commercial enzymes [20]. Therefore, in this study, the antioxidant peptides from *Pinctada martensii* meat were produced using simulated gastrointestinal hydrolysis, and after multistep purification, the structures of the peptides were identified using ultra-high-performance chromatography–tandem mass spectrometry (UHPLC-MS/MS), followed by the synthesis of the potential antioxidant peptides. The antioxidant activities of the peptides were verified using in vitro chemical assays and the HepG2 cell model, whose oxidative stress was induced by hydrogen peroxide (H_2_O_2_). The related mechanism of the peptides on H_2_O_2_-mediated oxidative stress was explored by determining the levels of ROS, lactate dehydrogenase (LDH), and glutathione (GSH); the activities of superoxide dismutase *(*SOD) and catalase (CAT); and the expression of key genes in the *Nrf2* signaling pathway in cells. The aim of this study was to provide technical and theoretical guidance for the use of *Pinctada martensii* meat in functional food development and to improve its utilization.

## 2. Materials and Methods

### 2.1. Materials

*Pinctada martensii* meat was obtained from Ronghui Co, Ltd. (Zhanjiang, China). Pepsin ( ≥400 U/mg), 2, 2′-azobis-2-methyl-propanimidamide (AAPH), and DCFH-DA were purchased from Sigma-Aldrich Co., Ltd. (St. Louis, MO, USA). Trypsin (2500 U/mg), Sephadex G-25, ABTS diammonium salt, and GSH were purchased from Yuanye Bio-Technology Co., Ltd. (Shanghai, China). Dulbecco’s Modified Eagle Medium (DMEM), 0.05% Trypsin–EDTA solution, fetal bovine serum (FBS), penicillin–streptomycin, TRIzol reagent, and the ReverseAid^TM^ First Strand cDNA Synthesis kit were purchased from Thermo Fisher Co., Ltd. (Waltham, MA, USA). K_2_S_2_O_8,_ absolute ethanol, K_3_[Fe(CN)_6_], trichloroacetic acid (TCA), FeCl_3_·6H_2_O, Trolox, DPPH, fluorescein sodium (FL), chymotrypsin (800 U/mg), and dimethyl sulfoxide (DMSO) were purchased from Macklin Biochemical Co., Ltd. (Shanghai, China). H_2_O_2_ (30%, *w*/*v*) was purchased from Dongzheng Co., Ltd. (Guangzhou, China). The Human hepatocellular carcinoma HepG2 cell line (ATCC: HB-8065) was purchased from American Type Culture Collection (ATCC, Manassas, VA, USA). The [3-(4,5-dimethyl-2-thiazolyl)-2,5-diphenyl-2H-tetrazolium bromide] (MTT) cell proliferation and cytotoxicity assay kit, the LDH level assay kit, the SOD assay kit (WST-1 method), the GSH assay kit, the total protein assay kit (with standard: BCA method) kit, and DEPC-treated water were purchased from Nanjingjiacheng Bio-engineering Co., Ltd. (Nanjing, China). The CAT assay kit was purchased from Beyotime Co., Ltd. (Shanghai, China). The 2 × TSINGKE^®^ Master qPCR Mix (SYBR Green I) kit was purchased from Tsingke Biotechnology Co., Ltd. (Beijing, China).

### 2.2. Preparation of HPM

*Pinctada martensii* meat was hydrolyzed according to the simulated gastrointestinal digestion protocol described before with slight modifications [1,2,3]. Briefly, *Pinctada martensii* meat was washed, dried, and blended with distilled water in a ratio of 1:2 (*w*/*v*). Then, the pH of the mixture was adjusted to 3.0 with 1 M HCl, which was followed by the addition of pepsin (2000 U/mL). After incubation at 37 °C for 2 h, the pH of the mixture was adjusted to 7.0, and then trypsin (100 U/mL) and chymotrypsin (25 U/mL) were added into the mixture. Followed by 2 h of incubation at 37 °C, enzymes were inactivated by boiling at 90 °C for 10 min. The digest was centrifuged at 8000 rpm for 20 min at 4 °C after being cooled to room temperature. The supernatants were named as HPM and stored at 4 °C. The method from GB/T 22492-2008 is used to measure the molecular weight of HPM [21]. Kjeldahl’s method was used to determine the protein content [22]. The degree of hydrolysate (DH) was calculated according to the following formula [23]:

DH (%) = (N_2_ − N_1_) / (N_0_ − N_1_) × 100% (1)

where N_0_ and N_1_ mean the assay of total protein and polypeptide in *Pinctada martensii* meat; N_2_ means the assay of the polypeptide in hydrolysates of gastrointestinal digestion.

### 2.3. Membrane Ultrafiltration

The sample of supernatants was separated sequentially using ultrafiltration with molecular weight cut-off (MWCO) membranes (Sartorius, Germany) of 10 kDa, 5 kDa, and 3 kDa respectively. Four fractions with MWs >10 kDa (HPM-1), 5–10 kDa (HPM-2), 3–5 kDa (HPM-3), and <3 kDa (HPM-4) were obtained. The obtained protein fractions were lyophilized and stored at −20 °C for further use.

### 2.4. Peptide Purification by Sephadex G-25 Gel Chromatography

The fraction after ultrafiltration exhibiting the highest antioxidant activity was further purified by Sephadex G-25 column (2.6 × 60 cm) gel filtration chromatography [24]. Briefly, a 10 mL sample was dissolved in distilled water with a concentration of 15 mg/mL after being filtered through a 0.22 μm pore-size water-phase membrane. The column was then eluted with distilled water at a flow rate of 1.2 mL/min. Each fraction was collected at 5 min intervals with a fraction collector that was monitored at 220 nm (UV755B, Shanghai Youke Instrument Co. Ltd., Shanghai, China). The pooled fractions were freeze-dried using a freeze-dryer (ALPHA 1–2 LDplus, Osterode, Germany) for further use.

### 2.5. Identification of Antioxidant Peptide by UHPLC-MS/MS

The eluted fraction that had the highest antioxidant activity was identified using ultra-high-performance chromatography–tandem mass spectrometry (UHPLC-MS/MS) (Thermo Fisher, Waltham, MA, USA) for peptide sequencing with an Accucore RP-MS column. In short, the mobile phase consisted of 0.1% (*v*/*v*) formic acid aqueous solution (A) and acetonitrile (B), and the elution phase was as follows: 0–4 min: 5% B, 4–6 min: 5–10% B, 6–30 min: 10–40% B, 30–34 min: 40–90% B, 34–40 min: 90% B, 40–42 min: 90–5% B, 42–50 min: 5% B. The column was set at a flow rate of 0.05 mL/min with a temperature of 40 °C. The mass spectrum was operated in full MS/DD-MS2 mode (positive-ion acquisition) with a primary resolution setting of 35,000, and the spectra were recorded with the mass/charge(m/z) range of 100–1500.

### 2.6. Prediction of Potential Antioxidant Activity of Peptides in Silico Methods

The potential antioxidant activity of peptides identified by UHPLC-MS/MS was selected using Peptide Ranker (http://distilldeep.ucd.ie/PeptideRanker/, accessed on 14 March 2022) and CPPpred software (http://distilldeep.ucd.ie/CPPpred/, accessed on 14 March 2022). Peptides were scored from 0 to 1, and the higher score means a higher potential to be bioactive and bioaccessibility in Peptide Ranker and CPPpred software, respectively [25,26].

### 2.7. Peptides Synthesis

The peptides selected as the potential antioxidant peptides from 4.6 were synthesized through solid-phase synthesis by Synpeptide Co., Ltd. (Nanjing, China). This process can be summarized as follows: the hydroxyl group of the C-terminal of the synthesized peptide chain was connected to a polymer resin with a covalent bond structure. The amino acid was first bonded to the solid-phase carrier as an amino component after the amino protecting group was removed, and a reaction with excessively activated carboxyl group components took place. The purity of the synthesized peptide verified through HPLC-MS was >95%. The synthesized peptides were stored at −20 °C for further use.

### 2.8. In Vitro Antioxidant Activity Assay

#### 2.8.1. DPPH Radical Scavenging Activity

The DPPH radical scavenging activity was measured according to the method described before [27]. Briefly, sample solution (1 mL) with different concentrations was mixed with 1 mL of DPPH (150 µM) in ethanol and incubated for 30 min in the dark. The absorbance was measured at 517 nm using a multifunctional microplate reader (SpectraMAX250, Molecular Devices, Sunnyvale, CA, USA). The control included DPPH and ethanol instead of sample, and the sample background containing sample and ethanol instead of DPPH was prepared as described above. GSH was used as a positive control. The results were expressed as the IC_50_ value (the concentration of sample required to scavenge 50% of DPPH radicals).

#### 2.8.2. ABTS Radical Scavenging Activity

The ABTS radical scavenging activity was measured according to the method described by Wang et al. [8]. The sample solution of different concentrations was added to 96-well plates at 20 μL and mixed with 180 μL ABTS^+^ working solution (diluting ABTS^+^ radical cation with ethanol until the absorbance values were in the range of 0.6–0.9). Blank control and positive control were distilled water and GSH, respectively. The reaction was well shaken and its absorbance was measured at 734 nm (Molecular Devices, USA). The results were expressed as the IC_50_ value (the concentration of sample required to scavenge 50% of ABTS radicals).

#### 2.8.3. Reducing Power

Reducing power was carried out according to the method described by Ahmadi et al. [28]. The samples (1 mL) were well mixed with 1 mL of 0.2 M phosphate buffer (pH 6.6) and 1 mL of 1.0% (*w*/*v*) K_3_[Fe(CN)_6_]. Then, the mixtures were incubated at 50 °C in a water bath for 20 min and centrifuged (3000 rpm, 10 min), followed by the addition of 1.0 mL of 10% (*w*/*v*) TCA to the sample solution. The supernatant thus collected (1.0 mL) was well mixed with 1.0 mL of distilled water and 0.2 mL of 0.1% (*w*/*v*) FeCl_3_ 6H_2_O. Next, it was allowed to stand at room temperature for 10 min. Color changes were monitored at 700 nm by a multifunctional microplate reader (Molecular Devices, USA). The higher the absorbance, the better the reducing power of the sample was.

#### 2.8.4. Oxygen Radical Absorption Capacity (ORAC) Assay

ORAC value was determined according to Huang et al. [29] with slight modifications. First, the Trolox standard was prepared as follows:  1 mM stock solution was diluted with 75 mM phosphate buffer (PBS, pH 7.4) to 6.25, 12.5, 25, and 50 μM working solutions, respectively. All the samples, FL, and AAPH solutions were prepared using PBS. Next, 20 μL of sample was mixed with 200 μL of fluorescein (96 nM). The mixture was then incubated at 37 °C for 10 min. After that, 20 μL of AAPH (119 mM) was added before the measurement of fluorescence (excitation and emission wavelengths of 485 and 538 nm, respectively). The fluorescence value was recorded every 4.5 min for 150 min (Molecular Devices, USA). GSH and PBS were used as the positive and negative control, respectively. The ORAC value was expressed as Trolox equivalents per gram dry weight (μmol TE/g DW) according to the area under the curve (AUC).

### 2.9. Cell Study

#### 2.9.1. Cell Culture

HepG2 cells were cultured in DMEM supplemented with 10% FBS and 1% penicillin–streptomycin solution and maintained in a 5% CO_2_ incubator at 37 °C. When the cells reached over 90% confluence, they were passaged and digested with a 0.05% trypsin–EDTA solution for further treatment.

#### 2.9.2. Evaluation of HepG2 Cell Viability

Cells were seeded in 96-well plates at a density of 2 × 10^5^ cells/mL and incubated for 24 h. To determine the cytotoxic effects of samples and H_2_O_2_, the cells were treated with various concentrations of peptides (24 h) and H_2_O_2_ (6 h), respectively. Vitamin C (500 μM) served as a positive control. The medium was removed after 12 h of peptide treatment, then the cells were induced to 2.00 mM of H_2_O_2_ for 6 h in order to detect the protective effects of peptides against H_2_O_2_-induced oxidative stress. After treatment, the medium was changed to 50 μL MTT and incubated for 4 h. The MTT was reduced to its insoluble formazan by cellular metabolic activity. The formazan crystals were dissolved in DMSO, and the absorbance was recorded at 570 nm, using a multifunctional microplate reader (Molecular Devices, USA). The MTT cell proliferation and cytotoxicity assay kit was employed to assess cell viability according to the manufacturer’s instructions.

#### 2.9.3. Protective Effect of S4 against H_2_O_2_- Induced Oxidative Stress in HepG2 Cells

##### Determination of ROS Level in HepG2 Cells

The scavenging of intracellular ROS by peptides was performed by the method reported by Wang et al. [30] with slight modifications. The cells (2 × 10^5^ cells/mL) were cultured in a 96-well black plate and incubated for 24 h at 37 °C and then treated with peptide (12 h) followed by treatment with H_2_O_2_ (2.00 mM, 6 h). Subsequently, the medium was removed, and the cells were washed with PBS. DCFH-DA (100 μL, 100 μM) dissolved in serum-free DMEM was added to each well and incubated for 30 min at 37 °C before washing with PBS again. Finally, the cells were cultivated in 100 μL serum-free DMEM and the fluorescence signals were detected with a fluorescent microplate reader (Molecular Devices, USA) at 37 °C. The program was set as follows: the excitation and emission wavelengths were 485 and 538 nm, respectively, and the measurement time was 30 min with the reading operation every 5 min interval. The results were expressed as the relative change of fluorescence intensity (%).

##### Determination of LDH Level in HepG2 Cells

The determination of LDH was conducted according to the method reported before [31] with slight modifications. HepG2 cells were seeded in 6-well plates at a density of 2 × 10^5^ cells/mL and incubated for 24 h at 37 °C. The cells were pretreated with peptides for 12 h before incubating with 2.00 mM H_2_O_2_ for a further 6 h. After treatment, the culture medium was collected and centrifuged for 4 min (1000 rpm, 4 °C) for determining the contents of LDH with the assay kit.

##### Determination of the Activities of SOD, CAT, and the Level of GSH in HepG2 Cells

The cells were pretreated with peptides for 12 h before incubating with 2.00 mM H_2_O_2_ for a further 6 h. Then the cells were lysed and centrifuged at (10,000× *g*, 4 min) to collect the supernatants after being washed with PBS. The activities of SOD and CAT and the level of GSH in the supernatants were assessed using assay kits, and the protein concentration of sample was estimated using a total protein assay kit. The results were then expressed as U/mg prot or μM/mg prot.

##### RNA Extraction and Real-Time Quantitative PCR (RT-qPCR)

HepG2 cells were treated with peptide (12 h) followed by treatment with H_2_O_2_ (2.00 mM, 6 h), and the gene transcriptions of *Nrf2, HO-1, NQO1, SOD*, *CAT,* and *GCLC* were determined by RT-qPCR. In brief, the total RNA of HepG2 cells with different treatments was extracted using TRIzol reagent according to the manufacturer’s instructions and dissolved into DEPC-treated water. RNA (2 μg) was reverse-transcribed into cDNA with the ReverseAid^TM^ First Strand cDNA Synthesis kit. To detect expression levels of the genes, RT-qPCR assays were performed in a BIO-RAD CFX48TM real-time system using the 2 × TSINGKE^®^ Master qPCR Mix (SYBR Green I) kit. The primer sequences were listed in Table 1. RT-qPCR was carried out under the condition of denaturation at 95 °C for 1 min, which was followed by 40 cycles of amplification. Data were analyzed by the 2^−ΔΔCt^ method.

### 2.10. Statistical Analysis

All the determinations were repeated in triplicate, and the results were expressed as mean ± standard deviation (mean ± SD). All data were analyzed by one-way ANOVA using SPSS 16 software (IBM, New York, NY, USA). Statistical significance was analyzed by the level of *p* < 0.05.

## 3. Results

### 3.1. Preparation and Antioxidant Activity of Pinctada Martensii Meat Hydrolysate (HPM)

After in vitro simulated gastrointestinal digestion, *Pinctada martensii* meat hydrolysate (HPM) had a molecular weight (MW) range of 22-69134 Da and an average MW of 1774 Da. The peptide yield was 55.34 ± 1.55%, and the degree of hydrolysate (DH) was calculated to be 31.51 ± 5.81%. As shown in Table 2**,** the IC_50_ values of ABTS, DPPH radical scavenging activities, and oxygen radical absorption capacity (ORAC) of HPM were 1.09 ± 0.02 mg/mL, 3.52 ± 0.13 mg/mL, and 463.91 ± 4.58 μmol TE /g DW, respectively, while the values for GSH were 0.04 ± 0.01 mg/mL, 0.08 ± 0.02 mg/mL, and 1118.56 ± 13.72 TE /g DW, respectively.

### 3.2. Purification of Antioxidant Peptides by Ultrafiltration

**Figure 1:**
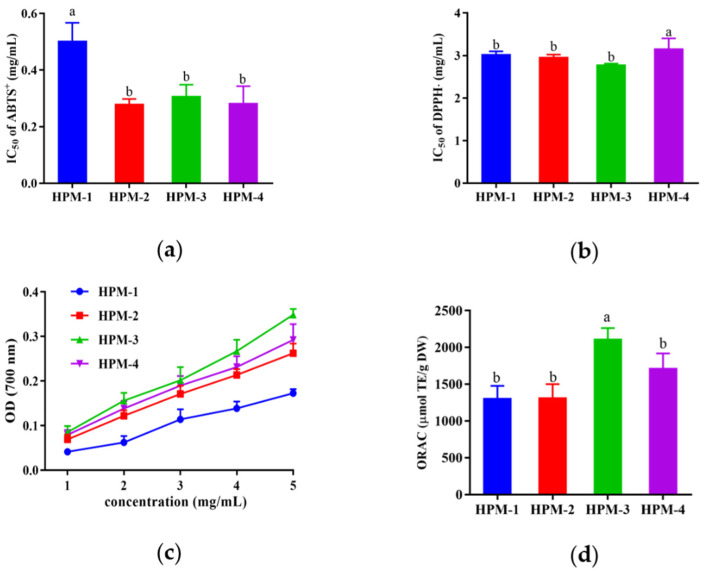
In vitro antioxidant assays of four fractions of HPM after ultrafiltration: (**a**) IC_50_ values of ABTS^+^ assay; (**b**) IC_50_ values of DPPH assay (**c**) reducing power assay; (**d**) ORAC assay. Data were expressed as mean ± SD (*n* = 3, *p* < 0.05). Different letters indicate significant differences (*p* < 0.05).

After ultrafiltration, four fractions with MWs >10 kDa (named HPM-1), 5–10 kDa (named HPM-2), 3–5 kDa (named HPM-3), and <3kDa (named HPM-4) were obtained, and various antioxidant assays (ABTS, DPPH radical scavenging assay, reducing power assay, and ORAC assay) were used to evaluate the antioxidative activities of the above four fractions. As illustrated in Figure 1, all of the four fractions could scavenge radicals. In the ABTS radical scavenging assay, HPM-2, HPM-3, and HPM-4 showed the lowest IC_50_ values (0.28 ± 0.02 mg/mL, 0.31 ± 0.04 mg/mL, and 0.28 ± 0.04 mg/mL, respectively), and there was no significant difference between them (*p* < 0.05). HPM-1, HPM-2, and HPM-3 had the lowest IC_50_ values of DPPH radical scavenging activity (3.01 ± 0.07 mg/mL, 2.96 ± 0.06 mg/mL, and 2.78 ± 0.03 mg/mL, respectively) without a significant difference (*p* < 0.05). Moreover, in the reducing power assay, HPM-3 and HPM-4 showed relatively higher OD values in a dose-dependent manner, which was followed by HPM-2 and HPM-1. In the ORAC assay, HPM-3 exhibited the highest ORAC value (2119.5 ± 98.76 μmol TE/g DW). In summary, the results suggest that HPM-3 had the strongest antioxidant capacity in vitro and was chosen for further study.

### 3.3. Purification of HPM-3 by Sephadex G-25 Gel Chromatography

**Figure 2:**
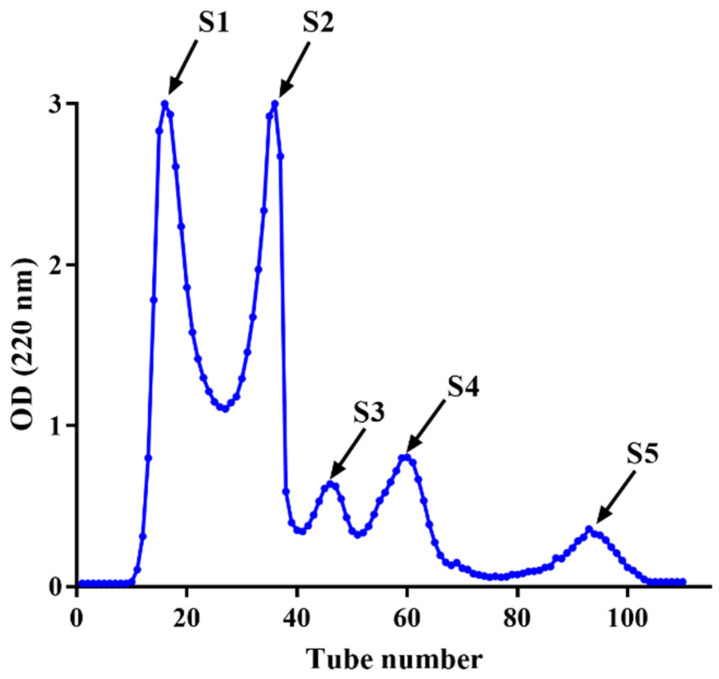
Purification of HPM-3 by Sephadex G-25 gel chromatography.

**Figure 3:**
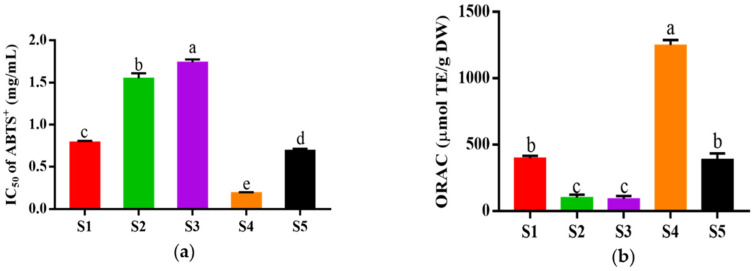
In vitro antioxidant activity of different fractions from HPM-3 separated by Sephadex G-25 gel chromatography: (**a**) IC_50_ values of ABTS^+^ assay of five fractions; (**b**) ORAC values of five fractions. Data were expressed as mean ± SD (*n* = 3, *p* < 0.05). Different letters indicate significant differences. (*p* < 0.05).

The fraction of HPM-3 was further purified with Sephadex G-25 gel chromatography. As shown in Figure 2, five fractions (S1–S5) were isolated. Among them, S4 exhibited the best ABTS^+^ radical scavenging activity with the lowest IC_50_ value of 0.19 ± 0.006 mg/mL (Figure 3a). In addition, the ORAC assay (Figure 3b) suggested that S4 displayed the highest ORAC value (1250.61 ± 39.26 μmol TE /g DW), whereas S3 showed the lowest ORAC value (91.49 ± 21.30 μmol TE /g DW). Hence, S4 showed the strongest antioxidant activity and was utilized to explore the protective effect on HepG2 cells against H_2_O_2_-induced oxidative stress.

### 3.4. Cytotoxicity of Peptide S4

**Figure 4:**
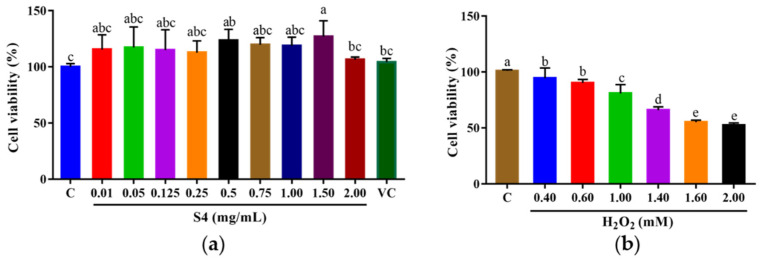
Cell viabilities of HepG2 cells treated with (**a**) S4 peptide or V_C_ and (**b**) H_2_O_2_ at different concentrations. “C” represents the control group. Data were expressed as mean ± SD (*n* ≥ 3, *p* < 0.05). Different letters indicate significant differences (*p* < 0.05).

The survival rate of HepG2 cells was analyzed by MTT assay after 24 h of treatment with the S4 peptide at different concentrations (0.01–2.00 mg/mL) or vitamin C (V_C_, 500 μM). As shown in Figure 4a, the cell viabilities of HepG2 cells ranged from 104.34% to 126.99% when treated with S4 at 0.01–2.00 mg/mL. The results suggest that S4 within tested concentrations was nontoxic to HepG2 cells. The following experiments were conducted under these concentrations.

After being cultured with H_2_O_2_ (0–2.00 mM) for 6 h, the cell viability was measured, and the results showed that the cell viability decreased with the increase in H_2_O_2_ concentration, and the trend showed a dose-dependent manner (Figure 4b). When the cells were cultured with 2.00 mM H_2_O_2_, the survival rate of HepG2 cells dropped to 52.59 ± 1.98%. Therefore, the oxidative stress model of HepG2 cells was established with 2.00 mM H_2_O_2_.

### 3.5. Protective Effect of Peptide S4 on HepG2 Cells against H_2_O_2_-Induced Oxidative Stress

#### 3.5.1. Effect of Peptide S4 on Survival Rate of HepG2 Cells against H_2_O_2_-Induced Oxidative Stress

**Figure 5:**
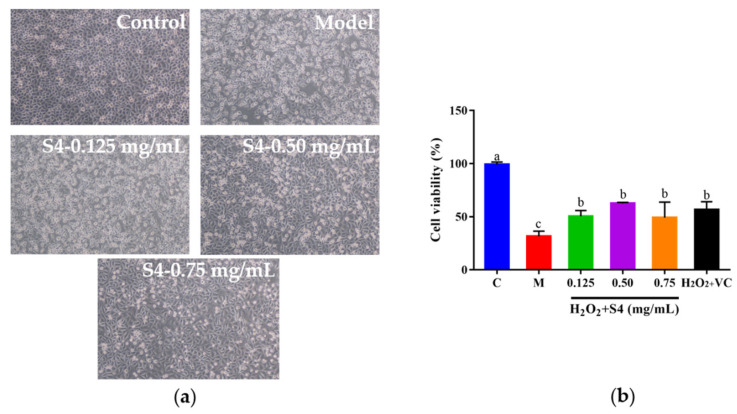
Effects of S4 at different concentrations on (**a**) cell morphology and (**b**) cell viability of H_2_O_2_-treated HepG2 cells. “C” represents the control group, and “M” represents the model group. Data were expressed as mean ± SD (*n* ≥ 3, *p* < 0.05). Different letters indicate significant differences (*p* < 0.05).

To explore whether S4 could protect cells from H_2_O_2_-induced oxidative damage, HepG2 cells were cultured with S4 at different concentrations for 12 h, followed by H_2_O_2_ treatment (2.00 mM, 6 h). As presented in Figure 5a, after H_2_O_2_ treatment, the cells became round and shrank, compared with the control group. When cells were treated with S4 before induced oxidative stress on cells, the morphology of the HepG2 cells was similar to the control group, especially in the medium- and high-concentration groups (0.50–0.75 mg/mL). Compared with the model group, the viabilities of H_2_O_2_-induced HepG2 cells increased to 60.14%, 61.52%, and 48.16%, respectively, with the concentrations of 0.125 mg/mL, 0.50 mg/mL, and 0.75 mg/mL. The results showed that the S4 peptide could enhance the survival rate of HepG2 cells under the circumstance of H_2_O_2_-induced oxidative stress.

#### 3.5.2. Effect of Peptide S4 on ROS and LDH Levels in HepG2 Cells

**Figure 6:**
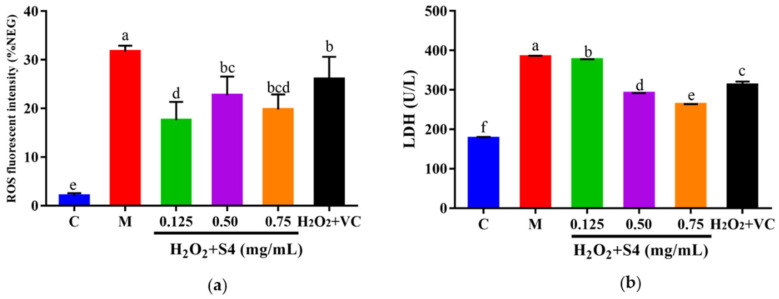
Effects of S4 on (**a**) ROS level and (**b**) LDH level of cells with oxidative stress. “C” represents the control group, and “M” represents the model group. Data were expressed as mean ± SD (*n* ≥ 3, *p* < 0.05). Different letters indicate significant differences (*p* < 0.05).

The ROS fluorescence intensity of samples was quantified with a microplate reader. As presented in Figure 6a, H_2_O_2_ treatment significantly increased intracellular ROS levels, triggering a 15-fold higher (*p* < 0.05) fluorescence intensity compared with that of the control group. However, the ROS level was notably (*p* < 0.05) reduced by treatment with S4 (0.125–0.75 mg/mL) or V_C_. Similarly, after 6 h of H_2_O_2_ exposure, a significant increase (2.2-fold, *p* < 0.05) in LDH level was observed in HepG2 cells, while treatment with S4 significantly decreased (*p* < 0.05) LDH levels in a dose-dependent manner (approximately 1.99% in low concentration, 22.19% in medium concentration, and 31.51% in high concentration, respectively), showing the better effect in reducing LDH levels than V_C_ (500 μM).

#### 3.5.3. Effects of Peptide S4 on the Activities of SOD and CAT, and the Level of GSH in HepG2 Cells

**Figure 7:**
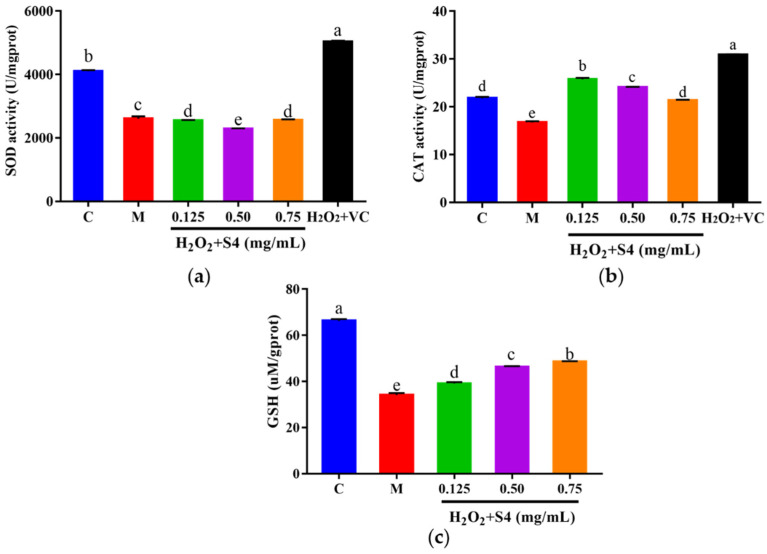
(**a**) SOD, (**b**) CAT activities, and (**c**) GSH level of HepG2 cells after S4 treatment. “C” represents the control group, and “M” represents the model group. Data were expressed as mean ± SD (*n* ≥ 3, *p* < 0.05). Different letters indicate significant differences (*p* < 0.05).

The intracellular antioxidant enzyme activities of SOD and CAT, as well as the level of GSH, were measured. As shown in Figure 7, a significant (*p* < 0.05) decrease in SOD, CAT, and GSH was observed in HepG2 cells exposed to H_2_O_2_ as compared with the control group (36.44%, 23.20%, and 48.48%, respectively). Treating with S4 did not increase the activity of SOD in oxidative HepG2 cells (Figure 7a). However, S4 significantly enhanced the activity of CAT compared to the model group (approximately 56.93% in low concentration). Significant dose–response effects of S4 were observed at GSH levels in HepG2 cells. When treated with 0.75 mg/mL of S4, the GSH level rose by 45.45% compared to that in the model group. These results indicated that S4 had positive effects on the activity of CAT and the level of GSH in oxidative-stressed HepG2 cells induced by H_2_O_2_.

### 3.6. Gene Expression of Nrf2 Signaling Pathway

**Figure 8:**
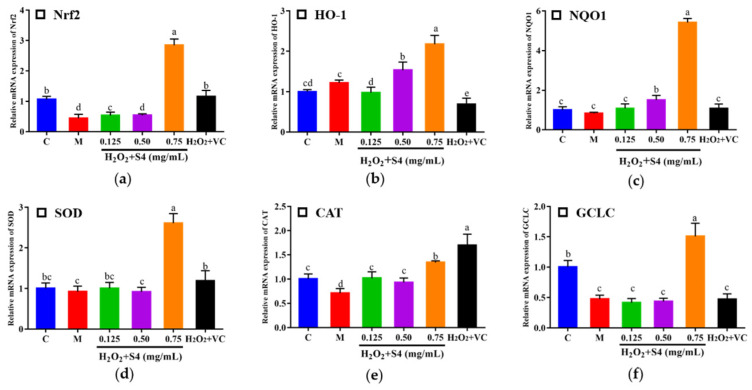
Effects of S4 on the relative expression of key genes in the *Nrf2* signaling pathway: (**a**) *Nrf2*; (**b**) *HO-1*; (**c**) *NQO1*; (**d**) *SOD*; (**e**) *CAT*; (**f**) *GCLC*. “C” represents the control group, and “M” represents the model group. Data were expressed as mean ± SD (*n* ≥ 3, *p* < 0.05). Different letters indicate significant differences (*p* < 0.05).

In this study, nuclear factor (erythroid-2-derived) related factor 2 (*Nrf2*), heme oxygenase-1 (*HO-1*), NAD(P)H: quinone oxidoreductase 1 (*NQO1*), glutamate-cysteine ligase catalytic subunit (*GCLC*), *SOD,* and *CAT* were determined by real-time quantitative PCR (Figure 8). After being treated with H_2_O_2_, the expressions of *Nrf2*, *CAT*, and *GCLC* decreased significantly compared with the control group (0.42-fold, 0.71-fold, and 0.48-fold, respectively). After S4 treatment at 0.75 mg/mL, the gene expressions of *Nrf2*, *HO-1*, *NQO1*, *SOD, CAT,* and *GCLC* were significantly increased compared with the model group (*p* < 0.05). The result suggested that treatment of S4 could activate the expression of *Nrf2* signaling-pathway-related genes.

### 3.7. Identification of Sequences from S4

**Figure 9:**
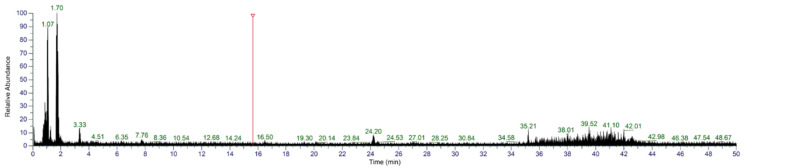
MS/MS spectra of S4.

To further identify S4, UHPLC-MS/MS was used. According to the first stage of mass spectrometry (Figure 9), the substances were mainly concentrated in the segments with low mass-to-charge ratio (100–250 m/z). The sequences of peptides were automatically deconvoluted using PepOS 1.0 Integrated Pep-tidomics Analysis Software (Wuyi University, Jiangmen, China), and a total of 65 peptides were identified**.**

### 3.8. Prediction of Potential Antioxidant Activity of Peptides in Silico Methods

The peptides were subjected to in silico analysis using Peptide Ranker (http://distilldeep.ucd.ie/PeptideRanker/, accessed on 14 March 2022) and CPPpred software (http://distilldeep.ucd.ie/CPPpred/, accessed on 14 March 2022), which predicted the potential bioactivity and cell-penetrating capacity, respectively, to further study the potential activities of peptides. As presented in Table 3, arginine–leucine (RL), arginine–glycine–leucine (RGL), proline–arginine (PR), phenylalanine–leucine–lysine–proline (FLKP), and leucine–leucine–arginine (LLR) showed a Peptide Ranker score ≥ 0.50, whereas RL, RGL, PR, and LLR obtained a CPP-pred score ≥ 0.50, indicating that these peptides probably had biological activities. Based on the above result, five peptides, RL, RGL, PR, FLKP, and LLR were selected to be synthesized through solid-phase synthesis for further analysis.

### 3.9. Cytotoxicity of the Synthetic Peptides

**Figure 10:**
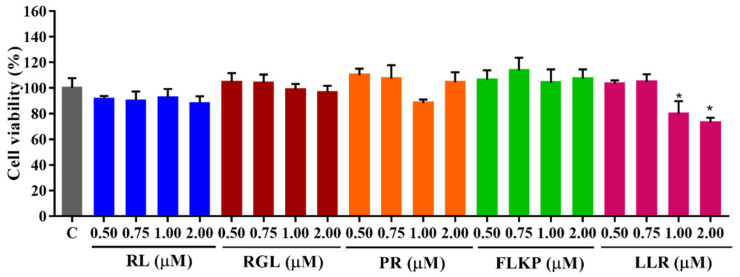
Cell viabilities of HepG2 cells treated with synthetic peptides. “C” represents the control group. Data were expressed as mean ± SD (*n* ≥ 3, *p* < 0.05). “*” indicated a significant difference from the control group (*p* < 0.05).

As shown in Figure 10, the survival rates of HepG2 cells remained above 80% after treatment with the four synthetic peptides (RL, RGL, PR, and FLKP) in the tested concentration (0.50–2.00 M) for 24 h. There was also no significant difference (*p* < 0.05) from the control group, which indicated they did not have toxic effects on HepG2 cells. After being treated with LLR, the cell viability decreased in a dose-dependent manner (1.00–2.00 μM of LLR), which indicated LLR was toxic to the HepG2 cells. Hence, the synthetic peptides were used for the further test with the above concentration except for the peptide LLR.

### 3.10. Protective Effect of the Synthetic Peptides of HepG2 Cells against H_2_O_2_-Induced Oxidative Stress

#### 3.10.1. Effect of the Synthetic Peptides on Survival Rates of HepG2 Cells

**Figure 11:**
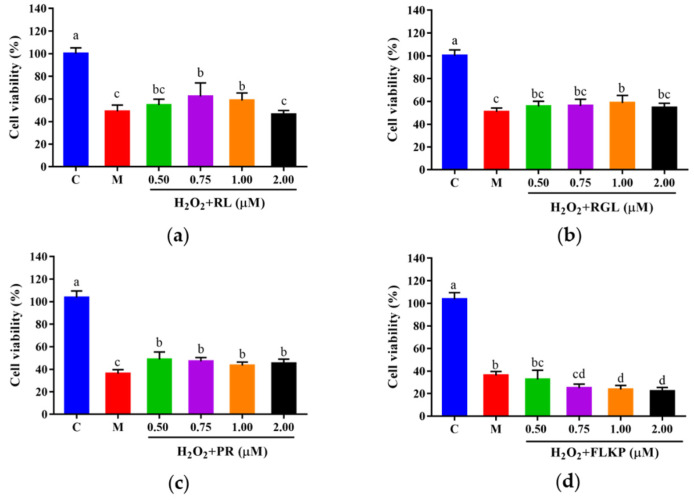
Cell viabilities of HepG2 cells with oxidative stress treated with synthetic peptides: (**a**) synthetic peptide RL; (**b**) synthetic peptide RGL; (**c**) synthetic peptide PR; (**d**) synthetic peptide FLKP. “C” represents the control group, and “M” represents the model group. Data were expressed as mean ± SD (*n* ≥ 3, *p* < 0.05). Different letters indicate significant differences (*p* < 0.05).

To explore whether the synthetic peptides could protect cells from H_2_O_2_-induced oxidative damage, HepG2 cells were cultured with the synthetic peptides at different concentrations (0.50–2.00 μM) for 12 h, followed by H_2_O_2_ treatment. Compared with the model group, the viabilities of HepG2 cells after treatment with the synthetic peptides (RGL, RL, PR) were significantly (*p* < 0.05) increased (Figure 11a–c). The synthetic peptides RGL, RL, and PR were able to increase the cell viability by up to 7.85%, 13.30%, and 15.21% (0.50 μM), respectively, when compared with the model group. The synthetic peptide FLKP could not increase the survival rate of oxidized HepG2 cells according to this study (Figure 11d). It was verified that the synthetic peptides RGL, RL, and PR could promote the survival rate of HepG2 cells in H_2_O_2_-induced oxidative stress except for the peptide FLKP.

#### 3.10.2. Effects of the Synthetic Peptides on the Levels of LDH, GSH, and SOD of HepG2 Cells

**Figure 12:**
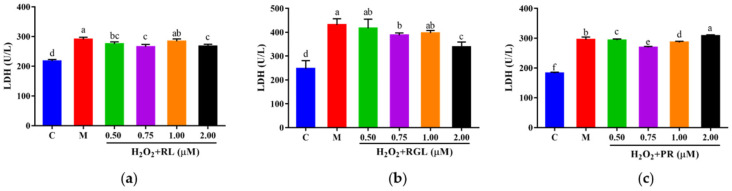
LDH level of HepG2 cells with oxidative stress treated with synthetic peptides: (**a**) synthetic peptide RL; (**b**) synthetic peptide RGL; (**c**) synthetic peptide PR. “C” represents the control group, and “M” represents the model group. Data were expressed as mean ± SD (*n* ≥ 3, *p* < 0.05). Different letters indicate significant differences (*p* < 0.05).

**Figure 13:**
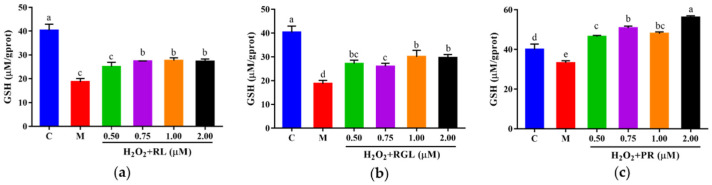
GSH level of HepG2 cells with oxidative stress treated with synthetic peptides: (**a**) synthetic peptide RL; (**b**) synthetic peptide RGL; (**c**) synthetic peptide PR. “C” represents the control group, and “M” represents the model group. Data were expressed as mean ± SD (*n* ≥ 3, *p* < 0.05). Different letters indicate significant differences (*p* < 0.05).

**Figure 14:**
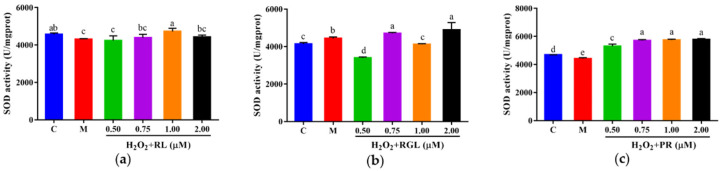
SOD activity of HepG2 cells with oxidative stress treated with synthetic peptides: (**a**) synthetic peptide RL; (**b**) synthetic peptide RGL; (**c**) synthetic peptide PR. “C” represents the control group, and “M” represents the model group. Data were expressed as mean ± SD (*n* ≥ 3, *p* < 0.05). Different letters indicate significant differences (*p* < 0.05).

The levels of LDH, GSH, and SOD were determined by assay kits. It was shown that after being treated with the synthetic peptides RGL, RL, and PR, the level of LDH decreased compared with that in the model group, and the synthetic peptide RGL exhibited the most significant decline (*p* < 0.05) (21.55% at 0.50 μM), with RL (8.68% at 0.75 μM) and RGL (8.91% at 0.75 μM) also showing some reduction (Figure 12). As shown in Figure 13, three synthetic peptides significantly increased the level of GSH in cells with oxidative stress, especially the peptide PR, in which there was a 68.57% decline compared with the model group. A similar effect was observed in the result of SOD activity (Figure 14), PR could significantly enhance the activity of SOD in HepG2 cells (30.75% over the model group). The result showed that three synthetic peptides (RGL, RL, PR) were able to reduce the H_2_O_2_-induced oxidative stress in HepG2 cells by reducing the LDH level, as well as increasing the levels of SOD and GSH.

## 4. Discussion

Enzymatic hydrolysis is the most common method for preparing peptides because it is eco-friendly and nontoxic [32]. Recently, two-stage enzymatic hydrolysis, known as simulated gastrointestinal digestion, has been utilized to hydrolyze protein substrates to obtain bioavailability peptides [23,33,34]. In this study, *Pinctada martensii* meat was used to prepare antioxidant peptides in this way. It was shown that HPM mainly consisted of small-molecule peptides and exhibited strong antioxidant activity in vitro. A similar result was also found in other studies. Zhang et al. [9] obtained antioxidant peptides from snakehead (*Channa argus*) soup by simulated gastrointestinal digestion, which had a high DPPH radical scavenging activity and Fe^2+^ chelating ability, respectively.

The HPM was separated using ultrafiltration to obtain four fractions with different MWs (>10 kDa (HPM-1), 5–10 kDa (HPM-2), 3–5 kDa (HPM-3), and <3 kDa (HPM-4), respectively). To compare their in vitro antioxidant activity, ABTS, DPPH radical scavenging activities, reducing power, and ORAC values were determined. It was shown that all fractions exhibited higher antioxidant activity than the crude HPM. In the ABTS radical scavenging activity assay, HPM-2, HPM-3, and HPM-4 showed the lowest IC_50_ values without a significant difference between them (*p* < 0.05). HPM-1, HPM-2, and HPM-3 had the lowest IC50 values of DPPH radical scavenging activity without a significant difference (*p* < 0.05). In the ORAC assay, HPM-3 showed the best ORAC value, and therefore it was used for further purification due to its potent antioxidant activity. According to our study, HPM-3 (3–5 kDa) had higher antioxidant capacity than HPM-4 (3 kDa), which was different from some reports that lower-MW ultrafiltration fraction had the highest antioxidant activity [13,35,36,37]. However, some studies have shown that there was no strict correlation between the molecular weight and antioxidant activity of peptides [12,38,39,40].

After being isolated from HPM-3 by Sephadex G-25 gel chromatography, Sample S4 was selected to evaluate the protective effect against H_2_O_2_-induced oxidative stress in HepG2 cells. It was reported that cell viability above 80% was considered noncytotoxic on cells [4]. Previous studies have reported that the optimal condition of H_2_O_2_ could be obtained to establish an oxidative damage model when the cell viability was about 50% [41]. Therefore, 2.00 mM H_2_O_2_ with an exposure time of 2 h was selected to establish the oxidative damage model.

The overproduction of ROS is known to induce oxidative stress, which is involved in various diseases [3,42]. Normally, LDH is mainly located in the cytoplasm and is released into the medium when the cell structure is damaged. Therefore, the degree of cell damage could be reflected through the measurement of LDH level [43]. Our study demonstrated the S4 peptide from *Pinctada martensii* meat could scavenge excessive ROS to reduce the degree of oxidative damage in HepG2 cells. Similar results could be observed in the study of the peptides from Octopus protein, which protected IEC-6 cells from H_2_O_2_-induced oxidative damage by reducing the generation of ROS and the leakage of LDH to improve cell viability [44].

Moreover, there are multifunctional antioxidant defense systems to eliminate excess ROS in cells and organs [45]. It contains some vital antioxidants, including CAT, SOD, and GSH. Our results suggest that the ability of S4 to protect HepG2 cells against H_2_O_2_-induced oxidative damage was associated with the ability to enhance the activities of CAT and the intracellular GSH level, but they did not increase SOD activity. Comparable results had been reported by Wang et al., who indicated that corn gluten peptide fractions had positive effects on the activities of the CAT and the GSH, but did not enhance the SOD activity [46].

*Nrf2* is the main regulatory factor of cellular redox reactions. It translocates to the nucleus when cells are stimulated by ROS, binding to antioxidant-related elements (ARE), thereby inducing the expression of phase II detoxifying and antioxidant enzymes, such as HO-1, NQO1, GCLC, SOD, and CAT [7,8]. Phase II detoxifying enzymes (HO-1, NQO1, GCLC, etc.) are vital for protecting cells from oxidative stress. For example, the rate-limiting step in the breakdown of heme to iron, carbon monoxide, and biliverdin is catalyzed by HO-1 [47]. NQO1 is a flavoenzyme that catalyzes the reduction of quinones to hydroquinones, which inhibits ROS production [48]. GCLC plays an important role in GSH biosynthesis [49]. From the results of RT-qPCR in the cells, the relative expression levels of *Nrf2, HO-1*, *NQO1*, *GCLC, SOD*, and *CAT* were promoted significantly after peptide treatment. Notably, the S4 treatment increased the level of SOD gene expression, which was contradictory to the decline in SOD activity in HepG2 cells.

Currently, in silico analysis is popular for the selection of bioactive peptides because of its time and cost efficiency [50]. To assess the potential activity and bioaccessibility of S4, in silico analysis was performed by Peptide Ranker and Cpppred software to predict the potential bioactivity and cell penetration behavior of the peptides [16]. In addition, the amino acid composition of the peptide also plays a significant role in the bioactivities of peptides [38,39,40]. Some hydrophobic amino acids such as glycine (Gly), leucine (Leu), and proline (Pro) were considered to have a positive effect on the antioxidant activity of peptides. These hydrophobic amino acids could act as hydrogen donors to transfer electrons to eliminate ROS and also increase the solubility of peptides in lipids, thereby accelerating interaction with the ROS and leading to stronger antioxidant activity [3,51]. Moreover, arginine (Arg) has been widely used in the inhibition of enzymatic browning [52,53,54] and lipid oxidation [55,56] because of its antioxidant capacity. Liu et al. [57] found that peptides Leu–Trp–Arg (LWR) had a strong free radical scavenging ability, which may have attribution to the role of Leu residues in the sequence. Xia et al. [20] discovered that Fraction M1 from *Pinctada martensii* mantle type V collagen exhibited stronger antioxidant activity than S1 from tilapia scale type I collagen due to the higher percentage of Gly and Pro. Moreover, it was observed that a number of di- and tri-peptides had better biological activity [58]. Combining the above studies, five peptides with potential antioxidant activity were chosen for solid-phase synthesis, and all of the five peptides contained one or more of these amino acids. The result showed that RL, RGL, and PR could protect the HepG2 cells from oxidative damage to increase the SOD activity and the GSH level, as well as decrease LDH leakage, which indicated these three novel peptides had antioxidant activities. Additionally, the synthetic peptides FLKP and LLR (PeptideRanker sore > 0.5) were unable to protect cells under oxidative stress, indicating that the use of Peptide Ranker and Cpppred software to screen bioactive peptides was limited. Compared with the synthetic peptides, S4 possessed the best effect on protecting cells from oxidative stress, which could be caused by the interaction between the peptides [59]. Therefore, it is essential to explore the structural mechanism of the synergistic effects among peptides in future studies.

## 5. Conclusions

In this study, simulated gastrointestinal digestion was utilized to prepare peptides from *Pinctada martensii* meat. The strongest antioxidant peptide (S4) was obtained after ultrafiltration and purified by Sephadex G-25 gel chromatographically. The result showed that S4 could protect HepG2 cells from H_2_O_2_-induced oxidative damage by reducing the generation of ROS and promoting the expression of key genes in the *Nrf2* signaling pathway to increase the activity of antioxidant-related enzymes. Additionally, five novel peptides were synthesized following the sequencing of the S4. It was found that synthetic peptides RGL, RL, and PR could reduce oxidative damage in HepG2 cells by increasing the intracellular antioxidant enzyme activity. The results would pave the way for exploring antioxidant peptides from *Pinctada martensii* meat by simulating gastrointestinal digestion.

## Figures and Tables

**Table 1:** Primer sequences.

Gene	Gene ID	Primer	Sequences (5′-3′)
*GAPDH*	2597	Forward	TCCACTGGCGTCTTCACCACCAT
		Reverse	GGAGGCATTGCTGATGATCTTGAGG
*Nrf2*	4780	Forward	GCTGATGGTACCCTGAGGCTAT
		Reverse	ATGTCCGCAATGGAGGAGAAGTCT
*HO-1*	3162	Forward	TGCCAGTGCCACCAAGTTCAAG
		Reverse	TGTTGAGCAGGAACGCAGTCTTG
*NQO1*	1728	Forward	GGAGACAGCCTCTTACTTGCCAAG
		Reverse	CCAGCCGTCAGCTATTGTGGATAC
*SOD*	6647	Forward	TGCAGGTCCTCACTTTAATCCTC
		Reverse	GCCACACCATCTTTGTCAGCA
*CAT*	847	Forward	ACCGTCATGGCTTAATGTTT
		Reverse	GATCTGTTGTGAAATCAGTGC
*GCLC*	2729	Forward	ACAAGAAATATCCGACATAGGAGA
		Reverse	CCATGTAAATATGATCCGGCTT

**Table 2:** In vitro antioxidant activities of HPM and GSH.

Antioxidation Activities	HPM	GSH
IC_50_ of ABTS^+^ (mg/mL)	1.09 ± 0.02	0.04 ± 0.01
IC_50_ of DPPH (mg/mL)	3.52 ± 0.13	0.08 ± 0.02
ORAC (μmol TE /g DW)	463.91 ± 4.58	1118.56 ± 13.72

**Table 3:** Identification of peptides in the fraction S4.

Sequences	PeptideRanker Score	CPPpred Score	Length	Mass (Da)
Arginine–Leucine (RL)	0.63	0.91	2	287.35
Arginine–Glycine–Leucine (RGL)	0.68	0.77	3	344.40
Proline–Arginine (PR)	0.79	0.81	2	271.31
Phenylalanine–Leucine–Lysine–Proline (FLKP)	0.79	0.28	4	503.63
Leucine–Leucine–Arginine (LLR)	0.52	0.88	3	400.50

## Data Availability

Data is contained within the article.
